# Ambient particulate air pollution induces oxidative stress and alterations of mitochondria and gene expression in brown and white adipose tissues

**DOI:** 10.1186/1743-8977-8-20

**Published:** 2011-07-11

**Authors:** Zhaobin Xu, Xiaohua Xu, Mianhua Zhong, Ian P Hotchkiss, Ryan P Lewandowski, James G Wagner, Lori A Bramble, Yifeng Yang, Aixia Wang, Jack R Harkema, Morton Lippmann, Sanjay Rajagopalan, Lung-Chi Chen, Qinghua Sun

**Affiliations:** 1 The Second Xiangya Hospital, Central South University, Changsha, Hunan, China; 2 Division of Environmental Health Sciences, College of Public Health, The Ohio State University, Columbus, Ohio, USA; 3 The Department of Environmental Medicine, New York University School of Medicine, Tuxedo, New York, USA; 4 Center for Integrative Toxicology and Department of Pathobiology and Diagnostic Investigation, Michigan State University, East Lansing, Michigan, USA; 5 Davis Heart and Lung Research Institute, The Ohio State University, Columbus, Ohio, USA; 6 Division of Cardiology, College of Medicine, The Ohio State University, Columbus, Ohio, USA

**Keywords:** air pollution, mitochondria, adipose, oxidative stress, inflammation

## Abstract

**Background:**

Prior studies have demonstrated a link between air pollution and metabolic diseases such as type II diabetes. Changes in adipose tissue and its mitochondrial content/function are closely associated with the development of insulin resistance and attendant metabolic complications. We investigated changes in adipose tissue structure and function in brown and white adipose depots in response to chronic ambient air pollutant exposure in a rodent model.

**Methods:**

Male ApoE knockout (ApoE^-/-^) mice inhaled concentrated fine ambient PM (PM < 2.5 μm in aerodynamic diameter; PM_2.5_) or filtered air (FA) for 6 hours/day, 5 days/week, for 2 months. We examined superoxide production by dihydroethidium staining; inflammatory responses by immunohistochemistry; and changes in white and brown adipocyte-specific gene profiles by real-time PCR and mitochondria by transmission electron microscopy in response to PM_2.5 _exposure in different adipose depots of ApoE^-/- ^mice to understand responses to chronic inhalational stimuli.

**Results:**

Exposure to PM_2.5 _induced an increase in the production of reactive oxygen species (ROS) in brown adipose depots. Additionally, exposure to PM_2.5 _decreased expression of uncoupling protein 1 in brown adipose tissue as measured by immunohistochemistry and Western blot. Mitochondrial number was significantly reduced in white (WAT) and brown adipose tissues (BAT), while mitochondrial size was also reduced in BAT. In BAT, PM_2.5 _exposure down-regulated brown adipocyte-specific genes, while white adipocyte-specific genes were differentially up-regulated.

**Conclusions:**

PM_2.5 _exposure triggers oxidative stress in BAT, and results in key alterations in mitochondrial gene expression and mitochondrial alterations that are pronounced in BAT. We postulate that exposure to PM_2.5 _may induce imbalance between white and brown adipose tissue functionality and thereby predispose to metabolic dysfunction.

## Background

Since air pollution has a major impact on public health for the general population, its health effects have been intensively investigated in recent years. Airborne particulate matter (PM) is a complex mixture of chemical and/or biological elements, composed of solid and liquid components that originate from vehicle exhaust, road dust, power plant stacks, forest fires, windblown soil, etc. In particular, airborne fine particulate matter (PM < 2.5 μm in aerodynamic diameter, PM_2.5_), i.e., PM in the fine and ultrafine ranges, has been implicated in the pathogenesis of cardiovascular disease and lung cancer [1-3].

Adipose tissue is now recognized as not only an energy reservoir for lipid storage, but also an active endocrine organ and an important regulator in glucose homeostasis. Adipose tissues are major actors in both obesity and the emergence of a cluster of associated diseases such as insulin resistance, type 2 diabetes mellitus (T2DM), cardiovascular diseases, and hypertension. There are at least two distinct types of adipose cells, white and brown adipocytes, with opposing effects on energy balance and body weight regulation. White adipose tissue (WAT) is highly adapted to store any excess energy as triglycerides, while brown adipose tissue (BAT), on the other hand, functions to dissipate chemical energy in the form of heat. Recently, A series of investigations have demonstrated that brown and white adipocytes are not sister cells, but rather that brown adipocytes are closely related to myocytes, and both originate from a common "adipomyocyte" precursor [4,5]. Among classical white adipocytes, two types may exist: the "genuine" white adipocytes, and "brite" (brown-in-white) adipocytes. Although "brite" cells do not possess the molecular characteristics of brown adipocytes, they possess the ability to express the uncoupling protein 1 (UCP1), which could mediate heat generation in brown fat uncoupling the respiratory chain and allow for fast substrate oxidation with a low rate of ATP production [6]. Moreover, brown adipose gene expression could be stimulated when mice are maintained at thermoneutrality and under conditions of cold acclimation [7,8].

Mitochondria play a key role in physiological process and are involved in the pathology of many diseases. Little is known about the physiological relevance of mitochondria in adipose tissue. It has been reported by Choo *et al *[9] that mitochondrial content and function in adipose tissue were reduced in the epididymal fat of type 2 diabetic mice, indicating a potential role for the disruption of adipose tissue mitochondrial content and function in T2DM. Previous studies have shown that fine particulate air pollution inhalation leads to insulin resistance, oxidative stress, alteration of vasomotor tone, vascular and visceral inflammation, adiposity, and atherosclerosis in apolipoprotein E knockout (ApoE^-/-^) mice and other several mouse models [10-14]. The ApoE^-/- ^mouse is particularly popular in research because of its propensity to spontaneously develop atherosclerotic lesions on a standard chow diet. It is used for studies of hyperlipidemia and atherosclerosis, and has been used extensively in understanding the mechanisms of lipoprotein metabolism and atherosclerosis. The ApoE^-/- ^mice are generated on a C57BL/6 background, and this model is highly susceptible to cardiovascular disease, overweight, insulin resistance, and the development of metabolic syndrome [10,13,15]. Although reports show that the function and expression of different adipose genes in white and brown adipose tissues [16,17], to our knowledge no study has investigated the impact of ambient air pollutants simultaneously in various of adipose depots. Therefore, the purpose of this study was to examine changes in white and/or brown adipose tissues in response to PM_2.5 _exposure in ApoE^-/- ^mice. We evaluated the role of PM_2.5 _exposure in inflammatory response, superoxide production, and alterations of mitochondria. Due to the functional differences in WAT and BAT including their vascularity, we hypothesized that PM_2.5 _exposure may have differential effects on these adipose depots. Thus, we systematically investigated the gene expression patterns in five different defined adipose depots: interscapular BAT (iBAT), mediastinic BAT (mBAT), inguinal WAT (iWAT), retro-peritoneal WAT (rWAT), and epididymal WAT (eWAT) [18,19] in response to PM_2.5 _exposure.

## Methods

### Animals

Four-week-old male ApoE^-/- ^mice from Jackson Laboratory (Bar Harbor, ME) were housed at constant temperature (22 ± 2°C) on a 12-h light/dark cycle. They were fed *ad libitum *on standard laboratory mouse chow and had free access to water. The investigation conforms to the Guide for the Care and Use of Laboratory Animals published by the US National Institutes of Health (NIH Publication No. 85-23, revised 1996), and the study protocols were approved by the Institutional Animal Care and Use Committee of Michigan State University and The Ohio State University under protocol #2008A006-R1.

### Exposure to ambient PM_2.5_

Animals were exposed to concentrated ambient PM_2.5 _or filtered air (FA) for 6 hours/day, 5 days/week for a total duration of 2 months in East Lansing, MI from June 7, 2010 to August 6, 2010. The concentrated PM_2.5 _in the exposure chamber was generated using a versatile aerosol concentration enrichment system (VACES) [10]. Inhalation exposures were conducted in one of Michigan State University's mobile air research laboratories (AirCARE 1) [20]. This laboratory is a 53-ft long, 36,000 pound semitrailer with approximately 450 ft^2 ^of interior laboratory floor space. Workspace within AirCARE 1 is divided into three work areas for: (1) atmospheric monitoring; (2) inhalation exposure systems for laboratory animals (rats or mice); and (3) biomedical laboratory for laboratory rodent anesthesia, surgery and necropsy, and sample storage. AirCARE 1 is certified by the Association for Assessment and Accreditation of Laboratory Animal Care (AAALAC). For the present study, AirCARE 1 was located on a Michigan State University research farm approximately 1 mile south of the main campus. The site is located over 1000 ft from a medium traffic roadway and 1,500 ft south of a lightly trafficked CSX railway. One interstate highway is located 2 miles south (I-96) and another 2 miles west (I-496) of the site, both of which carry over 25,000 vehicles daily. Michigan State University is located in East Lansing, MI (pop 46,420) in northern Ingham County, and is part of the Lansing Metropolitan Area (pop 453,603). Major emissions sources that could impact the exposure site are the T.B. Simon Power Plant, a 61 megawatt (MW) coal-burning facility located 1.2 miles northwest of the site. The Simon plant emits over 3,000 tons of SO_2 _and 1,300 tons of NO_x _annually. In downtown Lansing, approximately 4.5 miles west of the site is a 351MW coal burning power plant (Otto Eckert Station). The Lansing area also has a number of medium to light industries including automotive assembly plants (General Motors), steel (welding and fabricating) and metal processing facilities. Located in mid-Michigan, the site is also affected by regional emission sources in the Midwest, notably from the metropolitan Chicago area, industrial activities along Lake Michigan (e.g., Gary, IN), and coal burning power plants in the Ohio River Valley.

### Energy-Dispersive X-Ray Fluorescence (ED-XRF)

All PM samples for gravimetric and elemental analyses were collected on filters. Filter masses were measured on a microbalance (model MT5, Mettler-Toledo Inc., Highstown, NJ). Chemical composition was analyzed as described elsewhere [12,21].

### Dihydroethidium (DHE) staining

DHE (Invitrogen, Carlsbad, CA), an oxidative fluorescent dye, was used to detect superoxide (O_2_^-^), which binds to DNA in the nucleus and fluoresces red [22]. Briefly, fresh segments of the brown fat depots were frozen embedded in optimal cutting temperature (OCT) compound, and transverse sections (10 μm) were generated with a cryostat and placed on glass slides. Sections were then incubated in chamber with 10 μM DHE for 30 minutes at room temperature in a humidified chamber protected from light. Images were obtained with a fluorescent microscope. The excitation wavelength was 488 nm, and emission fluorescence was detected with the use of a 585 nm filter. Quantification of fluorescence intensity was determined by counting the number of positive stained nuclei in 10 random fields.

### Quantitative real-time PCR

Total RNA was isolated using TRIzol reagent as instructed by the manufacturer (Invitrogen, Carlsbad, CA), and reverse-transcribed to cDNA using the High Capacity cDNA Reverse Transcription Kit (Applied Biosystems, Foster City, CA). The quantitative real-time PCR analysis was performed with a light480 real-time PCR System (Roche Applied Science) following the standard procedure. Real-time PCR primer sequences including uncoupling protein 1 (*Ucp1*), peroxisome proliferator-activated receptor-γ coactivator 1-α (*Pgc-1α*), elongation of very long chain fatty acids 3 (*Elovl3*), type 2 iodothyronine deiondinase (*Dio2*), homeobox C9 (*Hoxc9*), insulin-like growth factor binding protein 3 (*Igfbp3*), dermatopontin (*Dpt*), and *β-actin *are showed in Table 1. Fold changes of mRNA levels were determined after normalization to internal control *β-actin *RNA levels.

**Table 1:** Primers used for real-time PCR

Gene	Forward primer (5' - 3')	Reverse primer (5' - 3')
*Hoxc9*	GCAGCAAGCACAAAGAGGAGAAG	GCGTCTGGTACTTGGTGTAGGG
*Igfbp3*	GCAGCCTAAGCACCTACCTC	TCCTCCTCGGACTCACTGAT
*Dpt*	CTGCCGCTATAGCAAGAGGT	TGGCTTGGGTACTCTGTTGTC
*Ucp1*	GGCCTCTACGACTCAGTCCA	TAAGCCGGCTGAGATCTTGT
*Pgc-1α*	GAAAGGGCCAAACAGAGAGA	GTAAATCACACGGCGCTCTT
*Dio2*	AAGGCTGCCGAATGTCAACGAATG	TGCTGGTTCAGACTCACCTTGGAA
*Elovl3*	GCCTCTCATCCTCTGGTCCT	TGCCATAAACTTCCACATCCT
*β-actin*	TGTGATGGTGGGAATGGGTCAGAA	TGTGGTGCCAGATCTTCTCCATGT

### Transmission electron microscopy (TEM)

Fat tissues were excised into small pieces (< 1 mm^3^) and fixed with 2.5% glutaraldehyde (0.1 M phosphate buffer, pH 7.4) for 3 hours. Each specimen was post-fixed in 1% osmium tetroxide for 1 hour and dehydrated through a graded series of ethanol concentrations before being embedded in Eponate 12 resin, sectioned at a thickness of 80 nm and stained by 2% aqueous uranyl acetate followed by lead citrate. The grids were then observed in a Technai G2 Spirit TEM (FEI Company, Hillsboro, OR). Quantitative analyses were carried out at a magnification of ×18500. An average of six to seven visual fields was evaluated for mitochondria analysis. The size of mitochondria was analyzed from randomly delineated in five to eight micrographs per group by NIH ImageJ software.

### Immunohistochemistry

Tissues were fixed overnight at room temperature in 4% formaldehyde, dehydrated in graded ethanol, followed by permeation in xylene and paraffin embedding. Five-micrometer-thick sections were deparaffinized and subjected to heat-induced antigen retrieval by incubation in Retrieve-all-1 unmasking solution (Signet Labs, Dedham, MA) for 15 minutes at 95°C. The slides were dipped in 0.3% H_2_O_2 _for 10 min to quench the endogenous peroxidase. After rinsing in phosphate buffered saline (PBS), the sections were incubated in 1% BSA/PBS for 10 minutes, followed by overnight incubation with rat anti-mouse F4/80 (AbD Serotec, Raleigh, NC) and rabbit anti-UCP1 (Abcam Cambridge, MA) at 4°C. Then the slides were rinsed and incubated at room temperature for 2 hours with appropriate horseradish peroxidase (HRP)-conjugated secondary antibodies. After the PBS rinsing, the stain was developed using Fast 3, 3'-diaminobenzidine tablet sets (D4293; Sigma, St. Louis, MO). The sections were then counterstained with hematoxylin and analyzed by a research microscope (Zeiss 510 META, Jena, Germany) with Metamorph V.7.1.2 software (Universal Imaging, West Chester, PA).

### Western blotting

Adipose tissues were homogenized in M-PER mammalian protein extraction reagent (Thermo Fisher Scientific), incubated on ice for 30 min, followed by centrifugation at 12000 *g *for 10 minutes at 4°C. The supernatant was collected and subjected to Western blot analysis. Protein concentrations were determined by BCA assay (Bio-Rad, Hercules, CA). Twenty microgram of protein was separated by SDS-polyacrylamide gel electrophoresis and subsequently transferred to PVDF membrane. After blotting in 5% non-fat dry milk in PBS-Tween 20 (PBS-T), the membranes were incubated with primary antibodies against β-actin (Sigma) or UCP1 (Abcam) overnight at 4°C, and then incubated with the appropriate horseradish peroxidase-linked secondary antibodies for 2 hours at room temperature. Finally, the membranes were visualized with an enhanced chemiluminescence kit (Pierce Biotechnology, Rockford, IL). Band density was quantified by densitometric analysis using NIH ImageJ software.

### Statistical analysis

Data are expressed as mean ± SEM unless otherwise indicated. The results of experiments were analyzed by unpaired *t *test using Graphpad Prism v4.0 (GraphPad Software, San Diego, CA). In all cases, *P *value of < 0.05 was considered as statistically significant.

## Results

### Exposure characterization

The mean (SD) daily PM_2.5 _concentration at the study site was 11.82 (6.71) μg/m^3^, while the mean concentration of PM_2.5 _in the exposure chamber was 96.89 μg/m^3 ^(approximately 8-fold concentration from ambient level). Because the mice were exposed for 6 hours/day, 5 days/week, the equivalent PM_2.5 _concentration to which the mice were exposed to in the chamber normalized over the 2-month period was 17.30 μg/m^3^. The mean elemental composition, as measured by energy-dispersive X-ray fluorescence (ED-XRF) analysis, is presented in Table 2.

**Table 2:** Elemental concentrations of PM_2.5 _particle during the exposure

	Ambient air		**Exposure to PM**_**2.5**_	
		
	Mean	**s.d**.	Mean	**s.d**.
**S**	1142.2	1045.7	10167.9	8038.0
**Al**	244.9	145.2	1297.9	571.0
**Si**	70.0	60.1	1166.9	918.7
**Ca**	37.9	37.8	631.7	519.5
**Fe**	27.5	20.5	403.5	211.6
**Mg**	64.6	73.3	365.0	281.7
**K**	49.3	24.0	358.4	216.1
**Na**	17.6	38.4	262.8	332.1
**P**	21.5	40.6	250.6	260.9
**Cl**	6.2	12.2	155.8	161.0
**In**	6.8	11.2	147.5	166.8
**Sb**	19.4	15.1	141.7	127.4
**Ba**	34.1	29.9	126.7	76.9
**Zn**	5.7	4.6	55.6	40.2
**Cs**	17.4	18.9	54.5	49.0
**Cd**	7.7	31.1	44.5	185.6
**Sc**	10.8	7.8	39.5	23.1
**Co**	10.0	7.3	35.2	21.9
**Br**	6.5	3.3	33.9	12.7
**Se**	8.1	5.6	28.2	15.0
**I**	5.7	15.7	28.2	55.6
**V**	8.5	5.0	27.2	12.9
**Mn**	4.1	6.0	24.9	18.8
**Ti**	1.8	2.4	22.2	20.0
**Pb**	4.4	6.4	21.5	21.8
**As**	4.3	5.2	18.2	14.9
**Rb**	4.5	2.7	13.7	7.2
**Ge**	3.5	4.7	11.9	11.7
**Cr**	1.4	2.2	11.5	24.7
**Sr**	2.4	2.0	11.3	6.2
**Ga**	2.0	3.7	11.1	10.7
**Te**	2.8	7.5	10.3	36.6
**Ni**	3.0	2.5	9.8	6.9
**Sn**	10.5	3.0	5.6	17.4
**Cu**	1.6	1.3	3.8	8.5

Note: unit, ng/m^3^. s.d., standard deviation.

### Superoxide (O_2_^-^) generation

**Figure 1:**
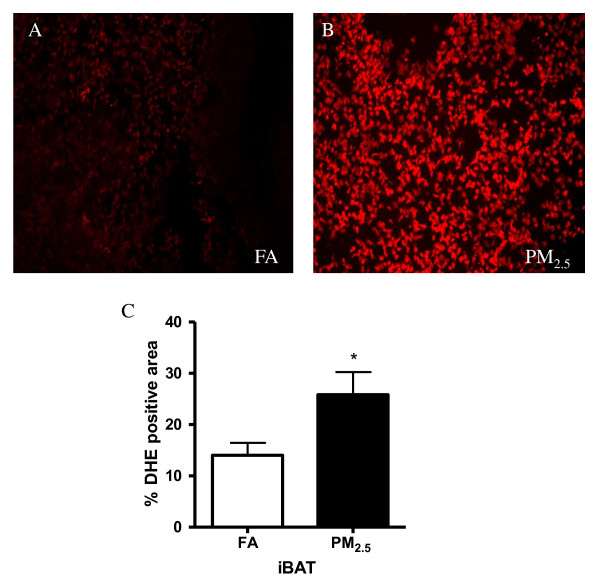
**Exposure to PM_2.5 _resulted in increased superoxide production in iBAT**. A. DHE staining of adipose tissue sections from the mice exposed to PM_2.5 _or FA for 2 months. Frozen iBAT sections were stained with DHE (10 μmol/L). The oxidative red fluorescence was analyzed by fluorescent microscope. B. DHE signals were quantified by the percentage of DHE-positive areas in 5 random fields. n = 8. *P < 0.05 *vs*. FA.

In order to test whether exposure to PM_2.5 _results in superoxide production in BAT, we performed dihydroethidium (DHE) staining on iBAT depots. As shown in Figures 1A-1C, O_2_^- ^production in the iBAT was markedly enhanced in the PM_2.5 _group compared with the FA group. O_2_^- ^that was accumulated in the iBAT of the mice exposed to PM_2.5 _was approximately 80% increase from FA-exposed controls.

### TEM analysis of in situ mitochondria

**Figure 2:**
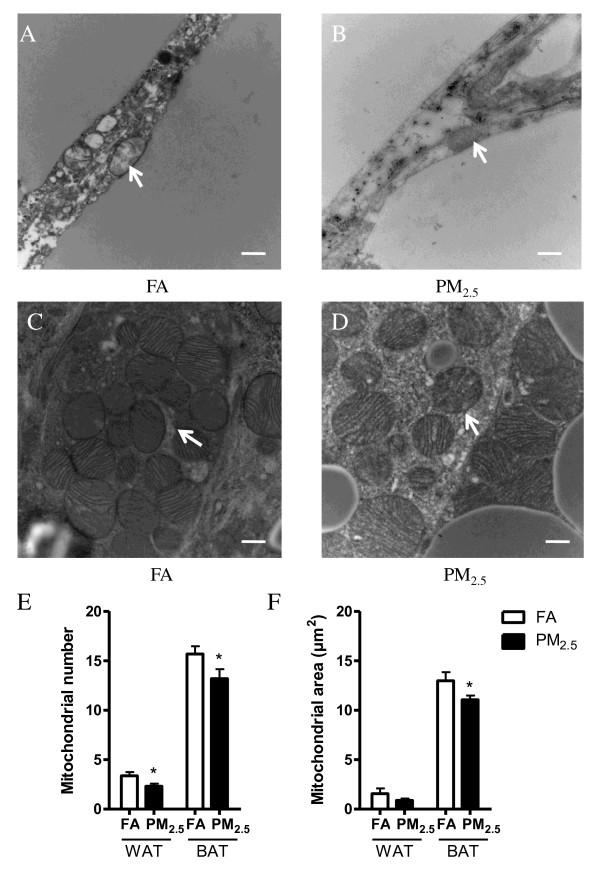
**The number and area of mitochondria in the eWAT and iBAT**. A-B. Representative TEM images of eWAT. C-D. Representative TEM images of iBAT (Arrows point to mitochondria). E: The analysis of mitochondrial number per field in the eWAT and iBAT. F: The analysis of mitochondrial area per field in the eWAT and iBAT. n = 4. *P < 0.05 *vs*. FA. Scale bars represent 500 nm in panels A, B, C and D.

To determine whether PM_2.5 _exposure affects mitochondria in WAT and BAT, transmission electron microscopy (TEM) was used in this study. Figure 2 shows representative TEM images of mitochondria in eWAT (Figures 2A and 2B) and iBAT (Figures 2C and 2D), respectively, and the analyses of mitochondria number (Figure 2E) and area (Figure 2F). In the PM_2.5_-exposed group, the mitochondrial number and area were significantly decreased in the iBAT when compared with the FA group. In addition, the mitochondrial number was also reduced in the eWAT in response to PM_2.5 _exposure, although we did not find significant differences in the mitochondrial area in these adipose depots.

### F4/80 and UCP1 expression

**Figure 3:**
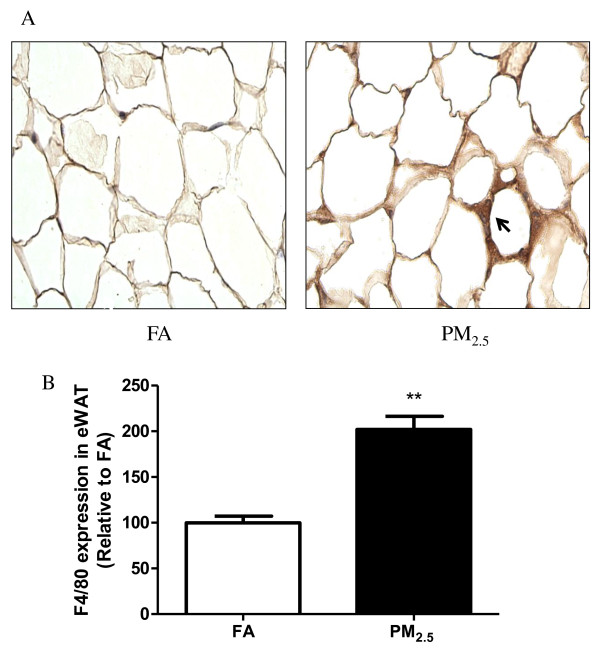
**PM_2.5 _exposure increases macrophage infiltration in the eWAT**. A. Immunochemistry for macrophage-specific marker F4/80 in sections of eWAT from FA- and PM_2.5_-exposed mice. B. Quantification of adipose tissue macrophages in eWAT. n = 4. **P < 0.001 *vs*. FA. Arrow shows F4/80^+ ^macrophages.

**Figure 4:**
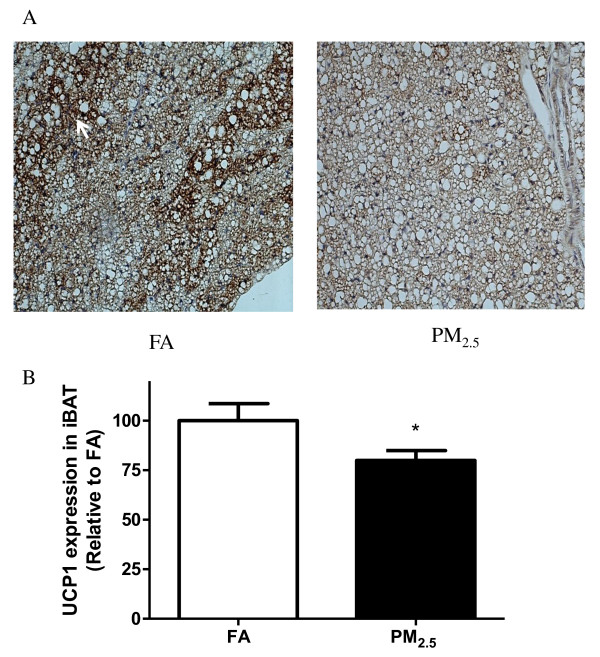
**Immunohistochemical examination of uncoupling protein 1 (UCP1) in the iBAT**. A. iBAT was stained by antibody against UCP1 and counterstained with hematoxylin. B. Quantification of UCP1 in iBAT. n = 8 *P < 0.05 *vs*. FA. Arrows show UCP1-positive brown adipocyte staining.

**Figure 5:**
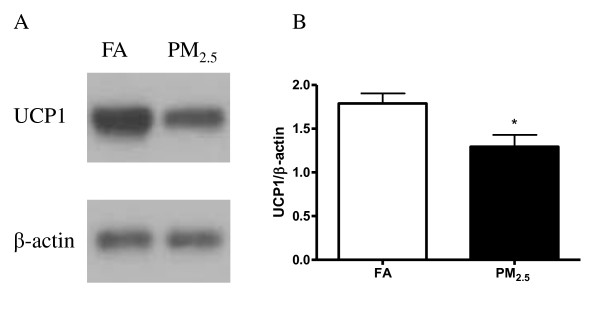
**PM_2.5 _exposure decreases UCP1 protein in the iBAT evaluated by Western blot**. A. Representative bands of FA and PM_2.5 _on UCP1 protein level in iBAT by Western blotting. B. Quantitative results of Western blotting of UCP1. n = 8. *P <0.05 *vs*. FA.

Adipose tissue macrophages (ATM), which are thought to represent key cellular mediators of adipose tissue inflammatory response and IR development, were examined in mice. As shown in Figure 3, PM_2.5 _exposure induced a marked increase in macrophage (F4/80^+ ^cells) infiltration in eWAT. Next, we analyzed the changes in uncoupling protein 1 (UCP1) in response to PM_2.5 _exposure. As shown in Figure 4, the data by immunohistochemical staining for UCP1 on the sections of iBAT (Figure 4) demonstrated that UCP1 expression was significantly decreased in the PM_2.5 _group. Western blotting data further confirmed down-regulation of UCP1 protein in the iBAT after PM_2.5 _exposure (Figure 5).

### BAT-specific gene expression

**Figure 6:**
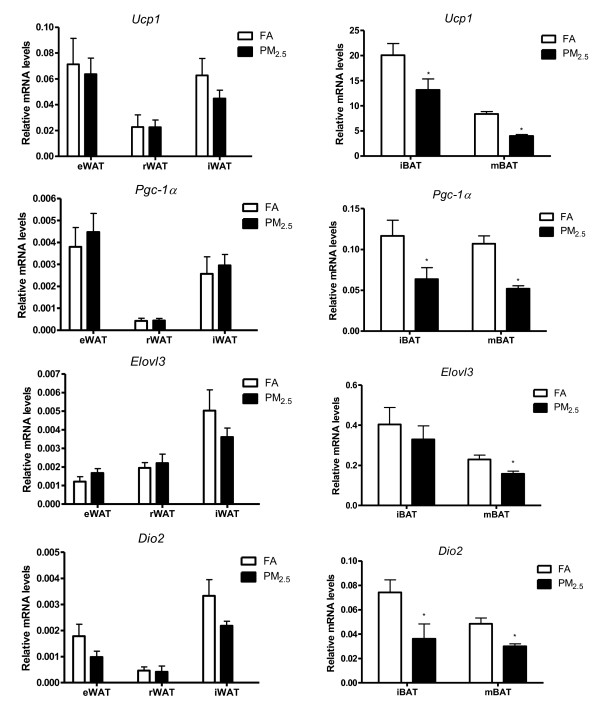
**Effect of PM_2.5 _exposure on brown adipocyte-specific gene (*Ucp1, Pgc-1α, Elovl3, Dio2*) mRNA levels in white adipose (eWAT, rWAT, and iWAT, left), and brown adipose depots (iBAT, and mBAT, right) by real-time PCR**. n = 8. *P < 0.05 *vs*. FA.

We next determined gene expression in different adipose depots in response to PM_2.5 _exposure, in terms of the expression of BAT-specific and WAT-specific gene profiles by real-time PCR analysis. UCP1 uncouples substrate oxidation and electron transport through the respiratory chain from ATP production. This is caused by an increased proton leakage over the inner mitochondrial membrane which dissipates the proton motive force as heat instead of ATP synthesis [23,24]. As shown in Figure 6, consistent with the fact that PGC-1α induces mitochondrial biogenesis and thermogenesis [25], its gene expression was marked in BAT compared with WAT (> 30-fold increased), while the level of *Ucp1*, which is almost classically associated with BAT function, was enriched more than 600-fold in BAT compared with WAT. The mRNA levels of the BAT-specific genes *Ucp1 *and *Pgc-1α *were however decreased in all defined adipose depots in response to PM_2.5 _exposure. The levels of down-regulation of both these genes were pronounced in iBAT and mBAT in comparison with the WAT depots. The gene expression of *Elovl3*, which is majorly expressed in BAT [26], was significantly decreased in mBAT by PM_2.5 _exposure. In addition, Dio2 may catalyze the conversion of T4 (thyroxin) into the active substance T3 (3, 3', 5-triiodothyronine), a process that occurs in all thyroid sensitive tissue but is particularly pronounced in BAT [27]. The mRNA levels of *Dio2 *were significantly reduced in both iBAT and mBAT in response to PM_2.5 _exposure. In this study, we did not find significant differences on BAT-specific gene expressions in the eWAT, rWAT and iWAT depots.

### WAT-specific gene expression

**Figure 7:**
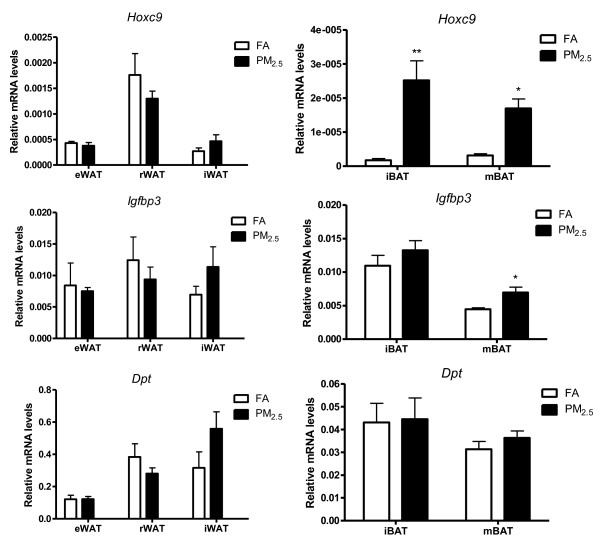
**Effect of PM_2.5 _exposure on white adipocyte-specific gene (*Hoxc9, Igfbp3, Dpt*) mRNA levels in white (eWAT, rWAT, iWAT, left), and brown adipose depots (iBAT and mBAT, right) by real-time PCR**. n = 8. *P < 0.05, **P < 0.001 *vs*. FA.

We also sought to determine if PM_2.5 _changed WAT-specific gene profiles in different depots. Igfbp3 is a family of six members important for insulin growth factor 1 (Igf-1) transport and storage in close proximity to the Igf-1 receptor (Igf1r), thereby facilitating Igf-1-mediated actions [28]. Hoxc9 belongs to the homeobox family of genes, and it is recognized as WAT-specific marker in primary adipocyte cultures [29]. DPT serves as a good gene marker for white adipogenesis and can be seen as a reference gene for the whitening phenomenon. As shown in Figure 7, the mRNA level of *Hoxc9 *was significantly higher in the iBAT and mBAT depots from PM_2.5_-exposed group than FA-exposed group. The mRNA level for *Igfbp3 *was also increased in mBAT in response to PM_2.5 _exposure. We did not observe significant differences in the gene expressions of *Hoxc9 *or *Igfbp3 *in the WAT, neither was the gene expression of *Dpt *in BAT or WAT.

## Discussion

In this study, we investigated the effects of inhalation exposure to PM_2.5 _on oxidative stress, inflammatory response, mitochondria and adipocyte-specific gene expression in adipose tissue depots. To our knowledge, this is the first study to systematically evaluate the effect of ambient PM_2.5 _on WAT and BAT specific genes in different adipose depots. There are several major findings in this study. First, exposure to PM_2.5 _resulted in oxidative stress in BAT. Second, exposure to PM_2.5 _induced changes consistent with reduced BAT functionality and a regression to a WAT phenotype [decrease in BAT specific genes (*Pgc-1α, Dio2, Ucp1*) and increase in WAT-specific genes (*Hoxc9 *and *Igfbp3*)]. This shift was not seen in WAT, when the same genes were analyzed. Finally, mitochondrial number was reduced in both eWAT and iBAT in response to PM_2.5 _exposure.

Recent studies have implicated PM_2.5 _in increased adipose inflammation and insulin resistance [11,12], and epidemiological studies indicate that obesity is associated with adverse health risks, such as hypertension and atherosclerosis [30]. PM_2.5 _has been shown to stimulate generation of reactive oxygen species (ROS) in cells due to its small diameters and large surface area [31]. To test if PM_2.5 _exposure could trigger ROS production *in vivo*, we examined the redox states in BAT. O_2_^- ^production was significantly increased in BAT in PM_2.5_-exposed mice compared with FA-exposed mice.

PM exposure has been demonstrated to cause mitochondrial damage in the pulmonary and cardiovascular systems [32,33], but little is known about the effects of PM_2.5 _on mitochondria in adipose tissues. In our study, we showed, by TEM measurement, that mitochondrial number was significantly decreased in response to PM_2.5 _exposure in both eWAT and iBAT, while the mitochondrial area was reduced in the eWAT depots as well. The possible mechanisms may include increased adipocyte membrane permeability or induced apoptosis caused by ROS [34].

BAT functional alterations in response to various stimuli have been investigated for many years but adaptation in BAT as a pathophysiological entity has only been recently investigated. Alterations in BAT function may influence propensity to obesity [35]. Indeed, prior studies suggest alteration of brown adipose gene expression in response to obesity and diabetes [36,37]. In addition to modulation of BAT functionality, there has been considerable interest in "brown-like adipose cells" in WAT. These so called "brite" cells are present in WAT as evidenced by the presence of UCP1 expressing cells in WAT. Studies in cell culture indicate that brown adipocytes and muscle cells share a common origin, which is distinct from white adipocytes [38]. A series of experiments has demonstrated that the UCP1 expressing cells constitute a subset of adipocytes ("brite" adipocytes) with a developmental origin and molecular characteristics [39]. The functional significance of these cells is not known, however; the presence of such cells in WAT raises important questions regarding potential regulatory pathways that may enhance or decrease "brown-fat" like functionality to WAT. In conditions of chronic cold exposure white-to-brown conversion meets the need of thermogenesis, while an obesogenic diet induces brown-to-white conversion, to meet the need of storing excess energy [40].

In this study, we found evidence of important changes in BAT in response to PM_2.5 _exposure. BAT expends energy through sympathetic nervous system-mediated non-shivering thermogenesis, where UCP1 is the key player [41,42]. UCP1 was significantly decreased in the iBAT. In addition, morphometric evaluation of TEM images indicated that mitochondrial number and size in BAT and the number (but not size) in WAT were reduced in response to PM_2.5 _exposure. Taken together, these data suggest that PM_2.5 _exposure may compromise the functionality of iBAT.

We found that PM_2.5 _exposure induces down-regulation of *Ucp1, Pgc-1α, Dio2 *and *Elovl3 *genes (change in *Elovl3 *seen only in mBAT) in classic BAT depots. On the other hand, WAT-specific genes *Hoxc9 *and *Igfbp3 *were up-regulated in brown adipose tissue, indicating brown adipocytes may potentially transform to a white adipose phenotype when stimulated by PM_2.5 _exposure. Interestingly, a similar shift was not seen in WAT suggesting that this phenotype is relatively specific for BAT.

Why these changes occur in BAT are beyond the scope of this paper, primarily due to limitations of sample size and tissue availability in each group. However, it is interesting to postulate that the increased vascularity of BAT may potentially relate to its vulnerability to air-pollution mediated effects. Future studies would need to be designed to provide significant insights into the roles and mechanisms of PM_2.5_-associated physiology and pathology.

In summary, our data demonstrate the important effects of PM_2.5 _exposure on oxidative stress and mitochondrial alterations in adipose tissues. These findings may have a significant impact on our understanding of the adverse effects of particulate air pollution on cardio-metabolic diseases, especially in the context of obesity and insulin resistance.

## Competing interests

The authors declare that they have no competing interests.

## Authors' contributions

ZX, XX, MZ, and AW performed the experiments and contributed to acquisition of data. ZX, XX, MZ, IPH, RPL, JGW, LAB, and YY analyzed the data and interpreted the results. MZ, IPH, IPH, RPL, JGW, and LAB contributed to PM_2.5 _exposure of the animals. The manuscript was written by ZX and XX and revised critically by YY, QS, ML, and SR. All authors read, corrected and approved the manuscript.
